# Disease Detection in Plum Using Convolutional Neural Network under True Field Conditions

**DOI:** 10.3390/s20195569

**Published:** 2020-09-28

**Authors:** Jamil Ahmad, Bilal Jan, Haleem Farman, Wakeel Ahmad, Atta Ullah

**Affiliations:** 1 Department of Computer Science, Islamia College, Peshawar 25000, Pakistan; jamil.ahmad@icp.edu.pk (J.A.); haleem.farman@icp.edu.pk (H.F.); 2 Department of Computer Science, FATA University, Kohat 26100, Pakistan; 3 Department of Agronomy, The University of Agriculture, Peshawar 25000, Pakistan; wakeel.ahmad@aup.edu.pk; 4 Agricultural Research Institute, Mingora Swat 19200, Pakistan; attaullahaup@yahoo.com

**Keywords:** disease detection, deep learning, plum

## Abstract

The agriculture sector faces crop losses every year due to diseases around the globe, which adversely affect food productivity and quality. Detecting and identifying plant diseases at an early stage is still a challenge for farmers, particularly in developing countries. Widespread use of mobile computing devices and the advancements in artificial intelligence have created opportunities for developing technologies to assist farmers in plant disease detection and treatment. To this end, deep learning has been widely used for disease detection in plants with highly favorable outcomes. In this paper, we propose an efficient convolutional neural network-based disease detection framework in plum under true field conditions for resource-constrained devices. As opposed to the publicly available datasets, images used in this study were collected in the field by considering important parameters of image-capturing devices such as angle, scale, orientation, and environmental conditions. Furthermore, extensive data augmentation was used to expand the dataset and make it more challenging to enable robust training. Investigations of recent architectures revealed that transfer learning of scale-sensitive models like Inception yield results much better with such challenging datasets with extensive data augmentation. Through parameter quantization, we optimized the Inception-v3 model for deployment on resource-constrained devices. The optimized model successfully classified healthy and diseased fruits and leaves with more than 92% accuracy on mobile devices.

## 1. Introduction

Agriculture has always been an important sector considering its economic impact on society, especially in developing countries. One of the significant challenges faced today is fulfilling the ever-growing requirements and demands for quality food products. Though different factors like crop diseases, climate change, and many others have a direct impact on food production [1], crop disease has been reported as one of the main sources of food losses that negatively impacts small-scale farmers. According to [2], around 80% of the agriculture is produced by small-scale farmers in developing countries, and food losses are much higher in these areas due to the lack of resources. As per the World Health Organization [3], more than 200 diseases are caused by unsafe food (viruses, bacteria, and chemicals) and around 600 million people get ill, in which 420,000 die every year. Further, it becomes more challenging for the developing countries to control and treat diseases on time so that the production and quality can be improved.

Fruit plant disease can have consequences not only on agriculture but also on the economy of the region. Apart from other food losses, around 10% of the food is demolished due to disease every year [4], which can even be high in developing countries depending on the type of crop, climate, and agricultural traditions. Some of the fruits are very vulnerable to diseases such as cherry, plum, and peach. Plum is considered a significant crop in Pakistan and recorded around 49,800 tons of production in 2017–2018 [5]. On average, a single tree production varies between 70 and 90 kg depending upon the variety and climate [6]. However, production is reduced by 10–15% due to various diseases, which affect productivity and quality. The most common diseases in plum fruit are brown rot, nutrient deficiency, and shot hole. In developing countries, farmers mostly rely on experts in pathology laboratories to detect and manage the diseases [4]. Detection of disease at its early stage is crucial to stop its spread to the entire plant and field, especially in fruit trees.

Disease detection in fruit plants has always been a challenge for farmers to look after the entire field by visiting each plant. In order to assist farmers in disease detection, different techniques based on artificial intelligence and computer vision have been proposed to identify the disease along with its severity level accurately. However, most of the experiments are performed in a controlled environment [7]. Essential factors such as image angle, scale, direction, and brightness are often ignored under such conditions. However, in a real environment, these have a direct impact on image processing and accuracy. Further, some applications such as Plantix [8] and GreenR [9] have been developed for disease identification by using cloud-based services. A farmer will send the captured image to the cloud for disease detection and expert opinion, which is time-consuming. However, in developing countries, internet connectivity is not available everywhere, and farmers in those regions are unable to use these applications. Moreover, a lightweight algorithm needs to be developed as it has to operate on smartphones. Technology needs to be transferred to the farmer so that the disease can be detected with possible treatments on-field to maintain crop health and improve production.

In this paper, the on-field disease detection and monitoring of plum fruit plants using a deep learning algorithm has been proposed to maintain plant health by minimizing further infection. It will help farmers detect early diseases in a plum fruit plant using smartphones in a real environment. In this regard, a dataset of normal and abnormal images is collected locally under true field conditions. The major diseases affecting plum fruit and leaves are brown-rot, nutrient deficiency, shot-hole (leaves), and shot-hole (fruit). All these diseases were covered in the collected dataset. Disease identification in plum fruit is a fine-grained classification problem where a healthy fruit is discriminated from an unhealthy one based on subtle differences in color and texture of the fruit. For instance, a healthy fully grown fruit can be confused with a fruit affected by shot-hole or brown-rot due to visual similarities to some degrees. Similarly, nutrient deficiency in leaves and shot-hole (leaf) have identical visual symptoms that make their detection very challenging. Using the dataset, we have trained and evaluated multiple convolutional neural networks (CNNs) to perform disease detection in images captured using mobile phones. While developing the proposed system, parameters related to images such as angles, scales, direction, and external environment are considered in order to train a robust detection model. The trained model is then optimized for deployment on resource-constrained mobile devices by quantizing the network parameters from 32-bit floating point (FP32) to 16-bit floating point (FP16). The final optimized model yields considerable accuracy and can be run conveniently on a mid-range device, eliminating the need to access cloud services.

The rest of the paper is organized as follows. The existing relevant literature is discussed in Section 2. The proposed method is covered in Section 3, while the results and discussion are thoroughly explained in Section 4. Finally, the paper is concluded along with future work in Section 5.

## 2. Literature Review

In the literature, both traditional machine learning and deep learning approaches have been widely used for disease identification in plants using image processing. Deep learning is considered one of the capable approaches not only to identify disease but that is used for disease severity as well. In [1], the publicly available PlantVillage [10] dataset of around 54,306 images in a controlled environment was used for disease identification in plants. The deep convolutional neural network was trained and two of the most popular architectures AlexNet and GoogleNet were considered. The model could recognize 14 different crops and 26 diseases with 99.35% accuracy. However, images with an upper side of leaves were evaluated, which cannot always be the case in real life. Disease can be at any part of the plant; therefore, datasets with many diverse images covering the whole plant may be required to accurately identify diseases. In [11], a deep learning method was used to automatically estimate the severity of plant disease by considering apple leaves from the PlantVillage [10] dataset. The authors have further annotated the healthy and black rot apple images that restrict the model only to one disease severity estimation. The authors were of the recommendation that the deep VGG16 model with transfer learning had an accuracy of 90.4%. However, the experiments were performed only on one disease, black rot, by considering only leaves. Further, most of the experiments in the literature were performed on leaves only and in a controlled environment, while the images in real-time in fields can be very challenging. Most of the factors that affect the quality of the captured images due to harsh field conditions were not considered.

Convolutional neural network (CNN) architectures have been widely used for plant disease identification [12]. The method was trained and tested by considering a public dataset containing 87,848 healthy and diseased leaves images. The dataset had a combination of images taken in a controlled and on-field environment. The 80/20 ratio was considered for training/testing. Authors achieved an accuracy of 99.53% in the classification of 20% of unseen images with VGG-16 architecture. The training and testing images were taken from the same database, which is commonly used in a classification-based model. However, the images taken in the field by farmers are more challenging, having different issues that are not considered in this approach. In [5], two approaches are adopted, the transfer learning method that is based on densely connected convolutional networks for plant disease detection on the server side, and a lightweight deep neural networks approach for end devices having constrained resources. The model performance was investigated using differently sized images at the input to come up with optimal image input sizes for different scenarios. The proposed model was trained and evaluated by considering a public dataset under a controlled environment. In [13], the authors proposed a deep meta-architecture approach to detect and locate infected regions in plants using three techniques. The region may be leaf, stem, or any other location. The system distinguishes the detected damaged plant section to be either an abiotic disorder or caused by an infectious agent (virus, bacteria, fungus, etc.). In other words, the system can detect whether the damaged region of the plant is because of noninfectious factors (low temperature, nutritional imbalance, etc.) or because of infectious microbes like viruses, bacteria, fungi, or nematodes. Authors have based the proposed deep meta-architecture of object recognition and classification mainly on three architectures, namely Faster Region-based Convolutional Networks (Faster R-CNN), Single Shot Multibox Detector, and Region-based Fully Convolutional Networks (R-FCN).

Authors in [7] proposed a two-stage algorithm tackling the problem of plant disease detection in complex background environments and severe environmental conditions. The authors claimed to have collected the largest image dataset of around 80 thousand images of 12 different species with 42 distinct classes. Augmentation of the dataset has been performed using traditional augmentation like geometrical and intensity transformation techniques and synthetic data generation from an existing dataset using a trained 5-layer deep convolutional generative adversarial network (DCGAN). For production of high-resolution images for the efficient training phase, the progressively growing GAN (ProGAN) technique was used to reconstruct high-dimensional images (typically 255 × 256) from smaller convolution blocks (128 × 64 × 3, 64 × 3, 64 × 128 × 3) in the discriminator network. This reconstruction of high-dimensional images was even more strengthened using a StyleGAN that amalgamates both ProGAN and neural style transfer. The trained StyleGAN combined with the backpropagation technique were used as a learning process for the diseased/infected portion of the leaf. The model, however, fails to produce desirable results with complex backgrounds, which is usually faced in real-time uncontrolled environments. Experiments were performed using the trained model on 18 different classes of the PlantVillage dataset, and these classes are supported on the tested PlantDisease dataset. For the AlexNet architecture, the model has achieved a validation accuracy of (99.13%, 81.24%) on (PlantVillage and PlantDisease Datasets), respectively. For DenseNet 201 and ResNet 152 architectures, the same validation accuracy was recorded to be (99.81%, 83.90%) and (99.75%, 82.92%), respectively.

Authors in [14] considered 1567 images of roughly 14 plant species from the PlantVillage database reflecting 79 different disease types. The images considered were captured with varying resolution and sizes on different sensor types: Smartphone, DSLR, and compact cameras. In the training phase, the controlled versus real field scenario images were of ratios 60% and 40%, respectively. The images were first manually subdivided considering only the leaf portion of the plant as the main target region for disease identification and classification with the backgrounds blacked out and the healthy portion of the total images considered as at least 20% to have contrast with lesions and the affected region. The authors based efficient CNN outcomes on the creation of a large and extended database (XDB) by using various transfer learning and augmentation techniques intelligently. However, the effort of database creation, claimed as several hundred hours, and the computation power required by GPUs are becoming a hindrance for real-time applications.

The authors in [15] studied the degradation problem in the training phase, which leads to significant reduction in accuracy where the depth of the network reaches its threshold. Authors have evaluated the use of a fine-tuned CNN for plant disease identification and classification on the freely available PlantVillage image dataset on different architectures including VGG 16, Inception-v4, ResNet, and DenseNets with a varying number of layers and respective image resizing constraints. Overall, 34,727 samples were taken into account with a validation set of 8702 samples and 10,876 images in the test set. The authors claimed that a fine-tuned 121-layer DenseNets outperformed other architectures on the ImageNet dataset for a higher training and validation accuracy of approximately 99.8% with high epochs. The authors claimed better results of ResNet and DenseNets over Inception-v4 and VGG, taking into account training time in training, computational requirements, efficiency obtained in transfer learning, convergence, weight requirement, and final accuracy with minimal loss.

## 3. Materials and Methods

Keeping in view the fact that the images will be captured under true field conditions where severe environmental lighting variations, sensory limitations of cheap mobile phone cameras, and the unfavorable image capturing environment may make it very challenging for simple image processing techniques to analyze [16], the whole process of image analysis was carefully designed to address all these issues. First, we prudently considered the various challenges being faced during the initial phase of the process. For instance, a farmer may capture the image of affected leaves or fruits at the top of the tree while standing on the ground. That will result in capturing a lot of background noise, loss of details due to the small scale of the objects of interest, and possibly motion blur. To cope with these challenges, the proposed method has been optimized to work effectively in true field conditions. Details of each step are provided comprehensively in the subsequent sections. 

### 3.1. Preprocessing

**Figure 1:**
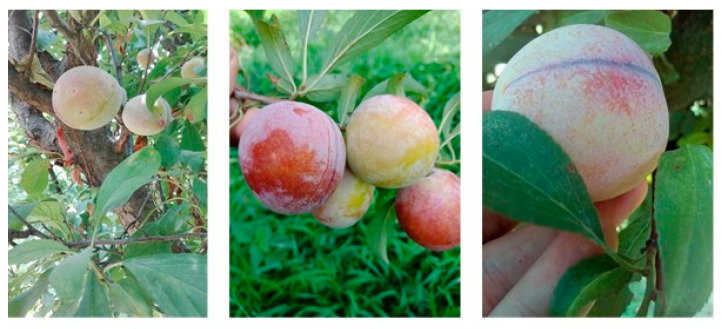
Scale variation in captured images.

In a typical end-to-end learning environment, there is very limited or no preprocessing step required. In most cases, the type of data available for training the end-to-end architecture is highly feasible for input, thereby eliminating the need for any preprocessing. In this case, the input had to be prepared so that it can be efficiently processed, and features can be effectively learned. Figure 1 shows the difference in scale for the objects of interest. For this purpose, extensive data augmentation was adopted where new images were generated from existing ones by varying the scale, position, and orientation. Details of the augmentations are provided in the subsequent sections.

### 3.2. Data Augmentation

Variations in scale, orientation, and position of the objects of interest in the images make predictions for CNNs very challenging. The network has to cope with all these variations in a robust manner. One of the most popular approaches we can use in situations like these is to use data augmentation. It is a process of generating more images from existing ones by applying similarity-preserving transformations. For our dataset, we decided to implement the following transformations to generate more images.

### 3.3. Geometric Transformations

Several geometric transformations, including scaling (3), rotation (5), and translation (image cropping at the center and four corners = 5), were applied on each image in the dataset to obtain 13 additional images from this transformation alone.

### 3.4. Contrast Adjustments

**Figure 2:**
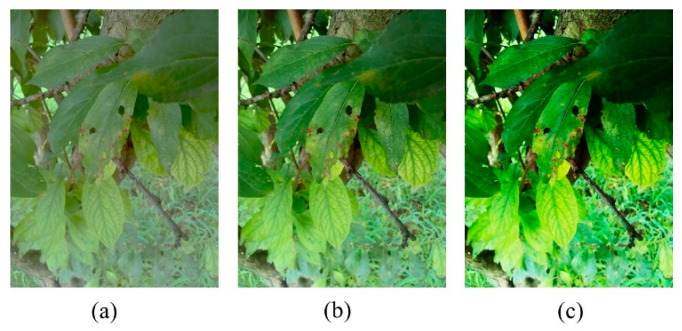
(**a**) Low-contrast, (**b**) medium-contrast, and (**c**) high-contrast images.

This transformation was applied to images in order to eliminate the effects of contrast variations in images due to varying lighting conditions in the field. The contrast stretching method defined in (1) was applied to images to introduce contrast variations in images, as shown in Figure 2.

(1) $$g(x,y)=\left\{\begin{array}{ll} a_{1}f(x,y), & f(x,y)<r_{1}\\ a_{2}(f(x,y)-r_{1})+s_{1}, & r_{1}\geq f(x,y)<r_{2}\\ a_{3}(f(x,y)-r_{2})+s_{2}, & f(x,y)\geq r_{2} \end{array}\right\}$$

where *g*(*x*,*y*) is the output; *f*(*x*,*y*) is the input pixel value; *r*_1_, *r*_2_, *s*_1_, and *s*_2_ are the user parameters to adjust contrast; *a*_1_, *a*_2_, and *a*_3_ are scaling factors for the various regions in grayscale and are defined as *s*_1_*/r*_1_, (*s*_2_ − *s*_1_)/(*r*_2_ − *r*_1_), (*L* − *s*_2_)/(*L* − *r*_2_), respectively; and *L* is the maximum gray level value.

### 3.5. Brightness Adjustments

**Figure 3:**
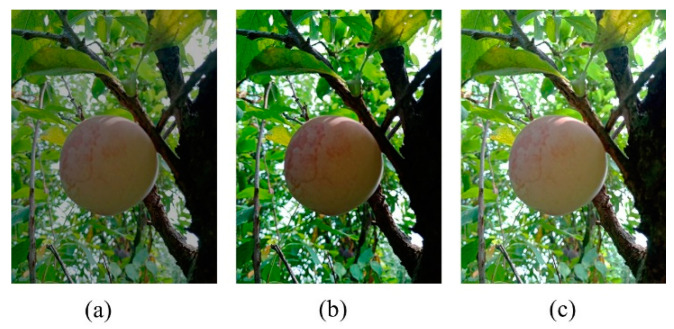
(**a**) Low-brightness, (**b**) medium-brightness, and (**c**) high-brightness images.

Environmental lighting variations can induce severe brightness changes in images. The gamma transforms (2) with varying amounts of gamma values for generating overly lit and under-lit images, as shown in Figure 3. Though the collected images did have such illumination-induced variations, this transformation helped improve the variations even further.

(2) $$g(x,y)=\alpha \cdot f{\left(x,y\right)}^{\gamma }$$

### 3.6. Saturation Adjustment

**Figure 4:**
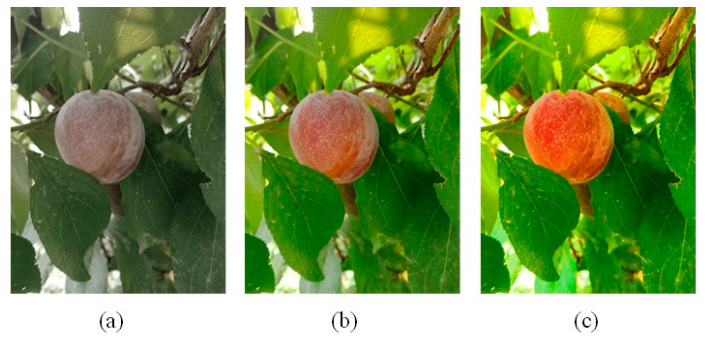
(**a**) Low-saturation, (**b**) medium-saturation, and (**c**) high-saturation images.

Mobile phone sensors vary widely based on the quality of the sensors. Low-cost mobile phones often come with mediocre cameras that have a poor quality of capturing colors. To reduce the effects of such sensory limitations, the saturation of images was slightly adjusted to generate high-saturation and low-saturation image samples. For this purpose, RGB images were first converted to the Hue-Saturation-Intensity (HSI) color model, and then after adjusting saturation, they were converted back to RGB. Some of the results of saturation adjustment have been shown in Figure 4.

## 4. Convolutional Neural Networks for Visual Recognition

Convolutional neural networks (CNNs) have been widely used for visual recognition tasks like image classification [17], object detection and recognition [18,19,20], image matching [21,22,23], image in-painting, and a variety of other similar challenging tasks. Its superior capability to automatically identify useful features from raw image data makes it a compelling choice for computer vision researchers [24]. As a result, significant research has been conducted on the development and use of CNNs for various challenging visual recognition tasks. Architectures ranging from straightforward to highly branched and sophisticated networks have been developed and shown to perform exceptionally well for the intended tasks. In this research, we experimented with several network architectures, including plane networks like AlexNet [24] and VGG-16 [25] and sophisticated networks like Inception [26] and Resnet [27]. Each of these architectures has its strengths and weaknesses. For instance, AlexNet is easy to develop and train; however, it provides lower accuracy due to the relatively shallow depth of the network by today’s standards. VGG-16, on the other hand, is an extensive and deep network, but it is tough to train and prone to overfitting. The Inception network is a very robust architecture having the built-in capability to process images at multiple scales. It is also more suited for deployment in mobile applications.

### 4.1. Network Architecture

**Figure 5:**
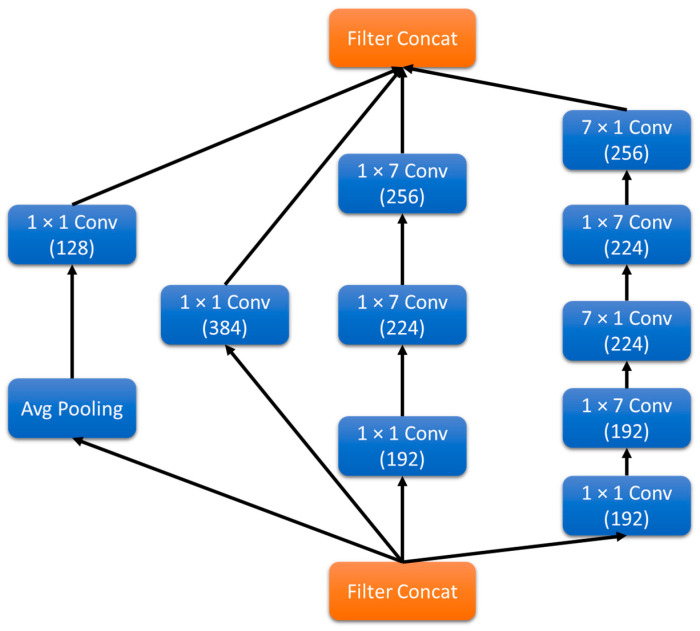
Inception module (Inception-v3).

Inception-v3 [26] is the third version of the famous GoogLeNet [28] architecture that won the ImageNet Large Scale Visual Recognition Challenge (ILSVRC) [29] in 2014. It is a powerful and robust CNN architecture developed by Szegedy et al. in 2015 to compete in the ILSVRC 2015, where it was the first runner-up. This network has improved inception modules and added a few more tweaks to obtain better performance and efficiency than the previous versions. Multi-scale processing at inception modules was a vital feature of these modules and a reason for their superior performance in so many tasks. With this version, they factorized the large convolutional filters into pairs of one-dimensional filters in the horizontal and vertical directions, as shown in Figure 5. That helped them achieve similar multi-scale analysis with a significantly reduced computational cost.

**Figure 6:**
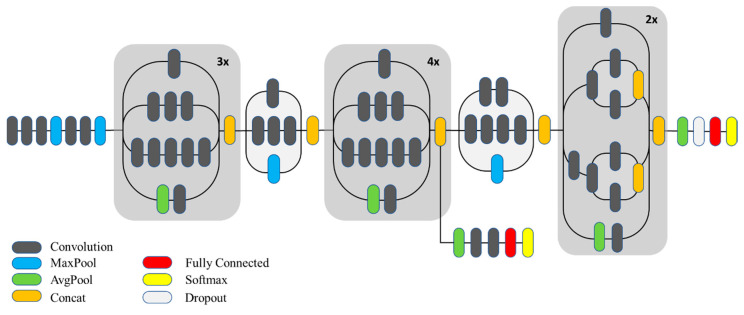
Inception-v3 architecture.

The network, as shown in Figure 6, inputs a 299 × 299 × 3 image and is processed initially by five convolutional layers where each layer applies several 3 × 3 kernels on the input. Afterward, a series of inception modules process the input before finally performing classification at the end by a fully connected layer. Unlike the previous versions, Inception-v3 has only one auxiliary classifier, which, according to the authors, acts as a regularizer. Between the blocks of inception modules, there is an efficient grid size-reduction block. Instead of applying to a pool for reducing the spatial dimensions of the input block, the activation dimension is first expanded using 1 × 1 convolutions, and then pooling operation is applied. This helps retain expressiveness in the model and avoids representational bottlenecks. All these tweaks make this architecture very robust for visual recognition tasks.

This network has been pre-trained on the ImageNet dataset to classify 1000 different types of objects in images. Due to its superior performance, integrated multi-scale processing, and computational efficiency, we opted to use this network to detect plum’s diseases. However, we had to either train this model from scratch or apply transfer learning in order to make this model recognize diseases in the target dataset. As this is a multi-class classification problem, the cost function we used with the network was categorical cross-entropy, as shown in Equation (3).

(3) $$l=-\sum_{k=1}^{K}\log(p(k))q(k)$$

where *l* is the loss, *K* is the number of class labels (in this case, *k* = 5), and *p*(*k*) is the probability of label *k*. Minimizing *l* results in maximizing the log-likelihood of the label, selected according to the ground truth distribution *q*(*k*). 

### 4.2. Network Training and Fine-Tuning

Training CNNs require large datasets and much computational power. However, training a robust and high-performance CNN requires a large but carefully designed dataset covering sufficient representative samples. The choice of a capable architecture for a particular problem is also key to achieving optimal performance. To this end, we trained a collection of different CNNs like AlexNet, VGG16, Inception-v1, and Inception-v3. These networks were trained on the target dataset to assess their performance. Although we were able to achieve around 86% performance with Inception-v3, we experimented with transfer learning in these models as well. The pre-trained models were previously trained on the ImageNet dataset. It is a large dataset of around 14 million images consisting of 21,841 different categories. It is essential to mention here that ImageNet contains 4486 synsets of plant, flora, or plant life, where each synset contains thousands of images covering a wide variety of plants. Though these CNNs were trained on one million representative images from 1000 categories, they can recognize a wide variety of plants as well. This makes CNNs trained on this dataset highly suitable for fine-tuning on other plant datasets. Such a model not only gets better at recognizing objects of interest but also performs better at rejecting irrelevant objects in the background.

Fine-tuning or transfer learning is the process of reusing parameters from pre-trained networks. Instead of randomly initializing network parameters, they are initiated with the parameters of pre-trained networks. This way, the network does not have to work hard to understand and learn essential visual features. Rather, the features extraction pipeline of ImageNet pre-trained networks is quite robust and diverse and can be effectively used to initialize networks for any visual recognition task. In addition to parameter initialization, we removed the last classification layer and added our layer according to the number of classes in the target dataset. The network was retrained for several epochs with a reduced learning rate so that most of the previously acquired knowledge was retained in the network. Fine-tuning moderately updates the parameters to achieve optimal performance on the target dataset. Settings for the various hyper parameters of various networks used in this study are provided in Table 1. Images in the dataset were resized according to the input requirements of each model for training and evaluation. In the case of fine-tuning, we were restricted to use the default input resolutions. However, in the case of newly trained models, higher-resolution inputs can be used. Unfortunately, doing so will increase the computational burden due to the increase in the number of convolution operations (due to larger activations maps) and the number of network parameters, which is not desirable in our case. Therefore, default input sizes were used with every network. Larger batch sizes were used for the relatively shallow AlexNet model. VGG16 is a very memory-intensive model; therefore, we had to set the batch size to 8. Due to the memory constraints, we could not set the batch sizes for both Inception models beyond 32. For the deep Inception-v3 model, the RMSProp optimization scheme was used to ensure faster convergence. For the rest of the networks, we used stochastic gradient descent with momentum (SGD-M). The learning rate for the newly trained models was set to 0.01 with an exponential decay. For fine-tuning, it was set to 0.005 in the beginning, which would decrease to 0.00005 in three steps. These parameters were set after thorough evaluation of these architectures.

## 5. Experiments and Results

In this section, performance details with both freshly trained CNNs and fine-tuned CNNs are provided in detail, along with an in-depth analysis of the convolutional layers’ sensitivity to affected regions in images.

### 5.1. Dataset Collection and Annotation

**Figure 7:**
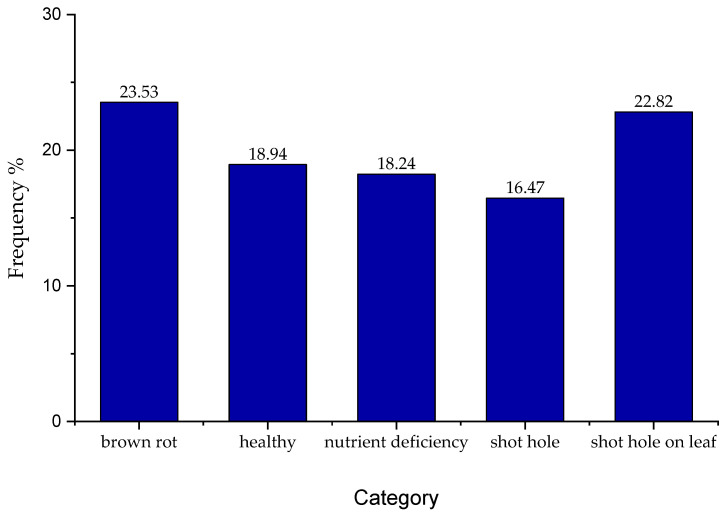
Class frequencies.

The dataset was collected from different areas of Khyber Pakhtunkhwa province in Pakistan. Mainly, the orchards in the Swat district inside Malakand Division were surveyed with a 15 day interval from June to September 2019. This allowed us to capture images of fruits at a variety of growth stages. The plum fruit usually takes about 2 months to reach maturity on a tree with an age between 5 and 12 years. Plum trees less than 5 years old were also covered in the dataset. Images were captured using a wide variety of mobile phones including a Samsung Galaxy S8 (16 MP) (Samsung, Seoul, Korea), Huawei (5 MP) (Shenzhen, China), Oppo A33 (8 MP) (Dongguan, China), and some (4 MP) cameras so that a varying degree of image quality could be obtained. Captured image resolutions were 3456 × 4608 (16 MP), 1920 × 2560 (5 MP), 2400 × 3200 (8 MP), and 2560 × 1440 (4 MP), respectively. Mobile cameras these days have become high-resolution ranging from 4 to 16 MP in the mid-range devices. Considerable detail was captured with the sensors used, which helped in producing augmented images with sufficient quality to enable effective training. A total of 5000 images were captured and annotated into five different categories, namely healthy, brown rot, shot hole fruit, shot hole leaf, and nutrient deficiency. Images were annotated on the basis of visual symptoms appearing on fruits and leaves, overall field conditions, and the presence of pathogens with the assistance of Plant Pathologist (Agricultural Research Institute, Mingora Swat, Pakistan). The class frequencies for various categories in the dataset are given in Figure 7. Classes were not perfectly balanced; however, the difference in frequencies is not substantial. Through data augmentation, 19 different versions of each image were generated, which resulted in a dataset having 100,000 images. The images were captured at different times of the day and under varying environmental lighting conditions.

The dataset was divided into training, validation, and test sets prior to data augmentation with ratios of 60%, 20%, and 20%, respectively. This allowed appropriate portions of both real and augmented images to be kept in all three sets so that the training and evaluation can be performed effectively. To test the robustness of the final trained model, some images of plum with healthy and diseased fruits were downloaded from the internet to test the performance of the model. A total of 100 images with 20 images in each category were used in this test.

### 5.2. Experimental Setup

All the experiments were conducted on a system running Microsoft Windows 10 (Redmond, WA, USA) powered by an Intel Core-i5 CPU (San Jose, CA, USA) equipped with 16 GB RAM. The deep learning experiments were carried out on an Nvidia GeForce GTX 1060 GPU (Santa Clara, CA, USA) with 6GB VRAM. This GPU has 1280 CUDA cores and is capable of training fairly complex networks. Google TensorFlow (Mountain View, CA, USA), BVLC Caffe (UC Berkeley, CA, USA), Nvidia DIGITS, and MathWorks MATLAB 2019b (Natick, MA, USA) were used for pre-processing, data augmentation, training, and evaluation. Different experiments were designed to evaluate the efficacy of the proposed method. In the first experiment, the overall detection and classification accuracy was measured. In further experiments, the convolutional activations of the trained model were analyzed to observe its sensitivity to diseased areas of the plants in the presence of background. This analysis helped us determine the effectiveness of the training process in automatically identifying the affected regions in the image. Results produced by the proposed method on test sets were verified by Plant Pathologist.

### 5.3. Sensitivity of Neurons in Deep Layers to Affected Fruit

**Figure 8:**
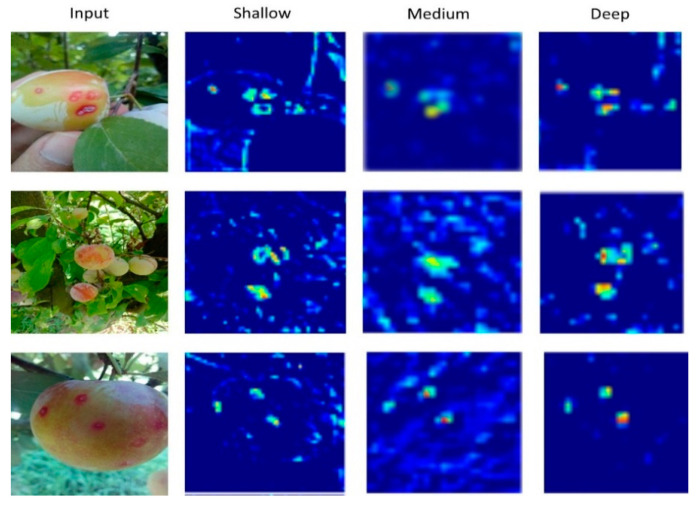
Activation response of various convolutional layers to lesions.

**Figure 9:**
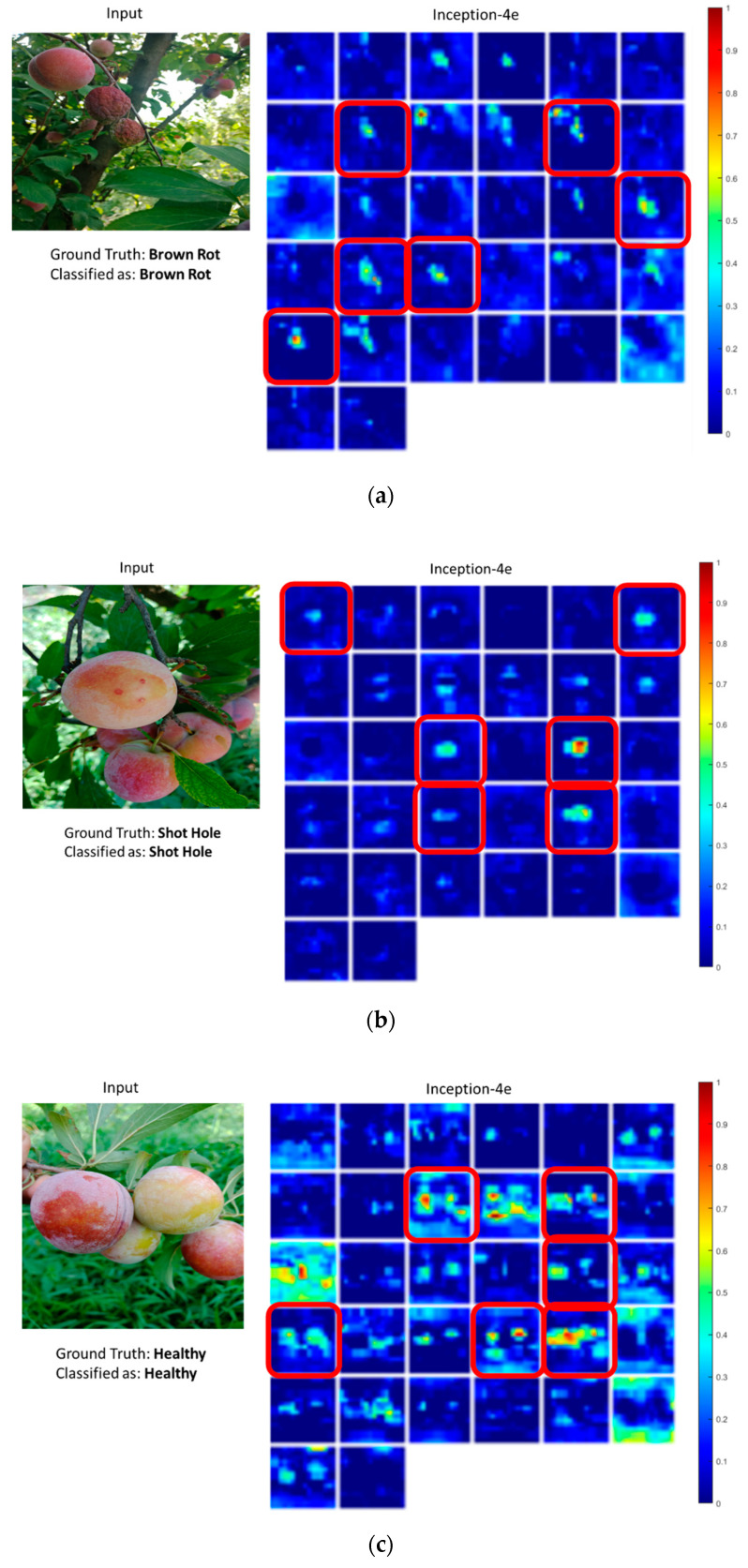

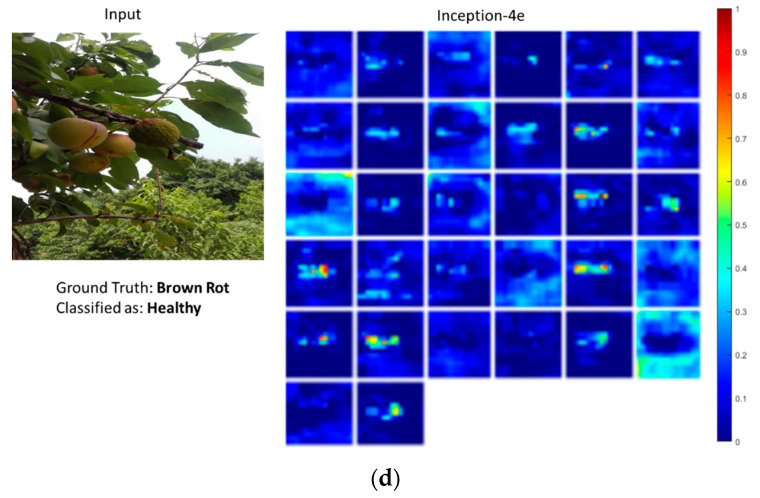
Activation maps output of a deep Inception module in Inception-v3 in cases of (**a**) brown rot, (**b**) shot hole, (**c**) healthy, and (**d**) brown rot. The sample in (**d**) has been incorrectly labeled as healthy. The red boxes indicate neurons with a higher sensitivity to affected fruit even in the presence of healthy fruit.

Neurons in deeper layers become sensitive to regions of interest in target images when fully trained. The study of activation maps reveals how and which neurons have become sensitive to the regions of interest. In this case, the regions of interest are the affected fruit or leaf in the image or the lesions on the fruits. In this regard, convolutional activation maps were analyzed by overlapping them onto original images. It was observed that, in most cases, the high activations of a small number of maps aligned with the affected regions of the image. Certain neurons in the convolutional network were producing high activations at those areas. Which defined the category label of the image, despite the presence of background clutter. This verifies that the CNN trained with the proposed method on the dataset has mainly learned useful features. Figure 8 shows some of the activation maps for their corresponding input images, taken from different layers of the network. It can be seen that the shallow layer activation maps were able to highlight the affected parts of the image, though not perfectly, which is reasonable as these layers typically learn low-level features. As we move deeper into the network, the activation maps get cleaner, and only the specific regions of the images are highlighted while rejecting the rest of the image (background). This analysis was carried out for all the models, where activation maps from specific convolution layers were overlapped onto the image. The results obtained from the Inception-v3 model were the most promising. Figure 9 shows activation maps from a deep convolutional layer (inception-4e) in Inception-v3. The highlighted maps indicate the affected regions in images with high activations, which shows that the network has adapted to the new dataset and has effectively learned to identify regions in the image leading to its final prediction. It is also important to note here that a unique set of neurons generate high activations for a particular category. For instance, in the case of healthy samples, a mostly unique set of neurons generate high activations unlike those in the case of brown-rot and shot-hole. Similarly, neurons sensitive to brown-rot remain inactive in all other cases. This shows that the neurons in the model have learned to recognize unique patterns in the images. In the case of samples (a, b, and c), correct predictions were made and the set of high activation maps have been highlighted in red. In the case of sample (d), the model was unable to correctly recognize the brown-rot. If observed closely, it becomes clear that the lighting condition is not ideal in this sample and the fruit has a greener color, which led to this confusion and misclassification.

## 6. Disease Detection Performance

In this section, the disease detection performance of both newly trained and fine-tuned CNNs is evaluated on the test set before and after data augmentation.

### 6.1. Performance with Newly Trained CNNs

All the models were trained for 30 epochs in order to evaluate their performance on the datasets prior to augmentation and after data augmentation. Using the dataset prior to data augmentation, we obtained results reported in Table 2. AlexNet achieved 53% accuracy on the test set, whereas the heavier VGG16 model was overfit due to the relatively smaller dataset being insufficient to tune the extremely large number of parameters. Both Inception models converged to some extent due to their superior architectures achieving 69% and 75% accuracies, respectively. Data augmentation helped significantly in model convergence and the achievement of much better performance. Performance improvements ranging from 9% to 12% were recorded with the use of the augmented dataset. As expected, the models yielded progressively better performance as we increased the depth and complexity of the networks, as shown in Table 3. AlexNet achieved 64.06% accuracy, which is the lowest in our experiments. With VGG-16, we were able to classify 78.68% of the test samples accurately. Inception networks performed very well in this experiment, owing to their capability of processing at multiple scales inside the inception modules. The recent version of the inception network yielded the best results of 86.81%.

### 6.2. Performance with Fine-Tuned CNNs

**Figure 10:**
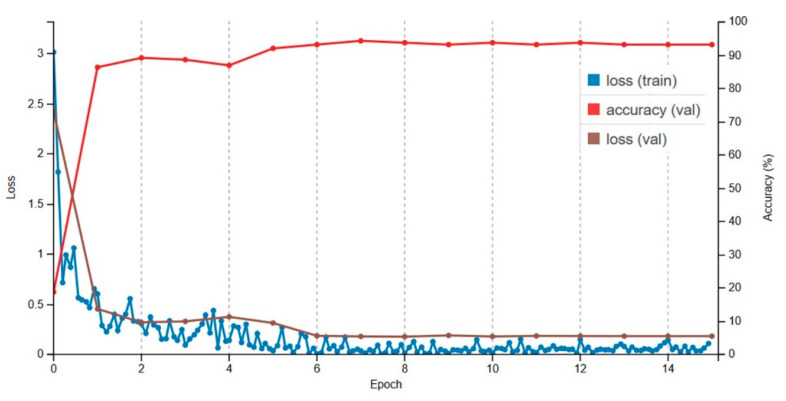
Fine-tuning progress of Inception-v3 with augmented dataset.

In this experiment, we fine-tuned all the networks for 15 epochs on the target dataset before and after data augmentation. Results reported in Table 4 and Table 5 highlight the benefits of using data augmentation with transfer learning. With fine-tuning on the augmented dataset, a considerable improvement in classification performance was noticed in all networks, particularly in AlexNet and VGG1-6. These architectures tend to benefit a lot from fine-tuning in this case due to the sheer number of parameters in these networks. On the other hand, significant improvements were also recorded in the Inception networks, where more than 90% accuracy was achieved with both networks. Inception-v3 resulted in a 94.34% accuracy after fine-tuning on the target dataset. Training progress of the fine-tuned Inception-v3 is provided in Figure 10. The optimal model was achieved at around the 7th epoch, which was used as the final model.

A class-wise prediction performance of the Inception-v3 model with the augmented dataset is provided in Table 6. It can be seen that the model is able to correctly classify samples of brown-rot, healthy, shot-hole (fruit), and shot-hole (leaf) categories with more than 90% accuracy. In the case of nutrient deficiency, the accuracy recorded was around 89%. Some of the samples in this category were misclassified as shot-hole (leaf) and vice versa. It is understandable keeping in view the visual similarities in both categories. Similarly, some healthy samples were incorrectly classified as brown-rot due to a certain degree of similarities in these categories. Overall, the model has lower false-positive and false-negative rates, which makes it suitable for use in this application.

**Figure 11:**
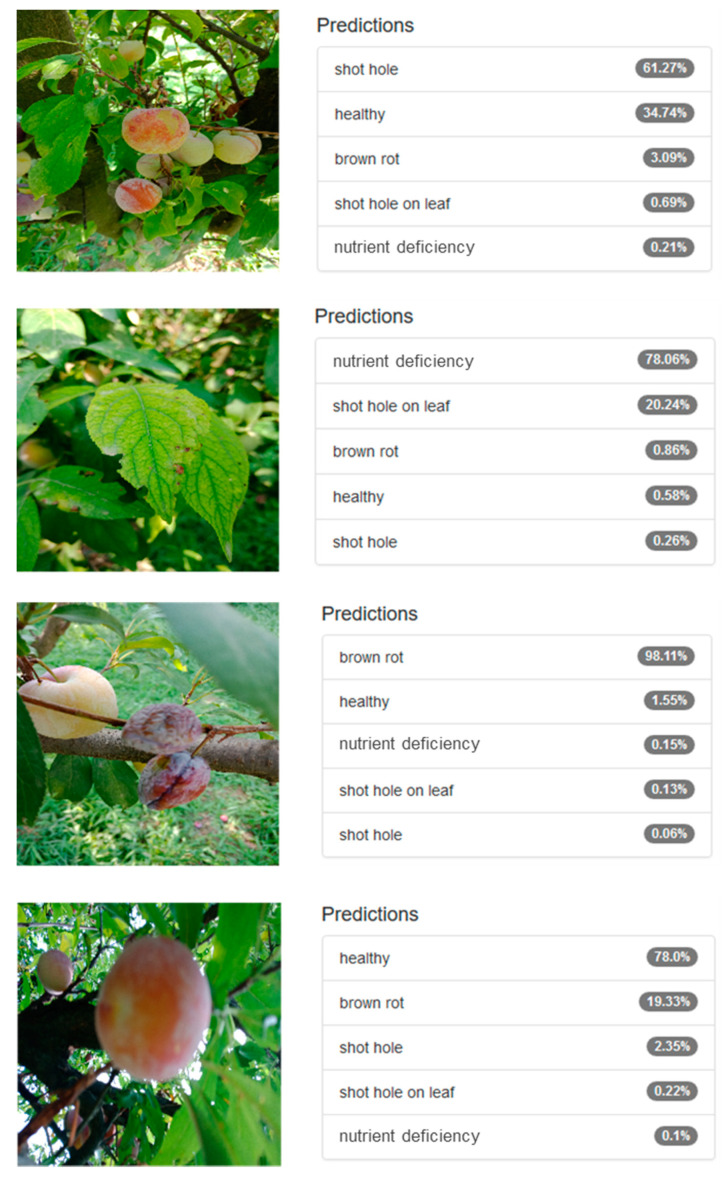
Prediction performance of Inception-v3.

Prediction performance on some of the test images is provided in Figure 11. It is interesting to note that the model could correctly classify the image even in the presence of background objects. For instance, in the first row, there is an unhealthy fruit in the image with shot-hole, which the model was able to classify correctly with 62% probability. However, looking at the predictions, it also indicates healthy fruit with 34.74% probability, keeping in view that the image contains healthy fruit in the image though at a smaller scale. Similar results can be seen for the rest of the test samples. In the last row, the model was able to correctly classify the image despite it being out of focus. In many such cases, accurate predictions were made despite variations in scale, translation, partial occlusion, and many other degradations, which exhibit the robustness of Inception-v3 for this problem.

### 6.3. Usage Feasibility of CNNs on Resource Constrained Devices

Mobile devices like phones and tablets are severely constrained in terms of memory and compute capabilities compared to full-sized PCs. The CPUs in these devices are usually far less capable, and having a reduced instruction set makes them fundamentally different from desktop CPUs. Consequently, a deep convolutional neural network deployed on a mobile device with full precision (FP32) will usually take several times longer during inference. To address this problem, CNNs with reasonable requirements for compute power are usually selected and deployed on such devices (e.g., MobileNet [30], ShuffleNet [31], etc.). In addition to this, a parameter quantization approach is also utilized, which transforms network parameters from a 32-bit floating point (FP32) to a 16-bit floating point (FP16) or even integer (INT8), which not only reduces the memory requirements but also reduces computational requirements considerably, at the cost of minor performance degradation. In our case, we chose to use the Inception-v3 model and convert it to FP16 for better efficiency on mobile devices. The Google TensorFlow Lite convertor tool [32] was used to perform the conversion from FP32 to FP16 in order to make it feasible for deployment on mobile devices. The problem under consideration does not require real-time inference, yet it is better to deploy an efficient architecture for smooth overall operation on a wide range of devices. A summary of the computational and memory requirements for various models is provided in Table 7. It can be seen that the quantized version of the trained Inception-v3 yielded considerable performance while requiring far less memory and time for inference. The model was tested on a variety of devices equipped with a Qualcomm Snapdragon-845 (high-end, 8-core ARM CPU) (San Diego, CA, USA), Qualcomm Snapdragon-801 (low-end, 4-core ARM CPU), and Qualcomm Snapdragon-730 (mid-range, 8-core ARM CPU). Both Inception models were very efficient in inference on these devices when quantized to FP16, requiring less than 150 ms on Inception-v1, and less than 500 ms on Inception-v3. The AlexNet and VGG models were not converted, being low performers and requiring huge memory and computations.

### 6.4. Robustness of the Proposed Method

The quantized model was evaluated on a new set of sample images, which were downloaded from the internet in order to assess its robustness in terms of capturing device, environmental illumination, and image resolution. In this regard, a set of 100 images having 20 images in each category were used to evaluate the model performance. The confusion matrix obtained as a result of these evaluations is provided in Table 8. Though the performance is slightly reduced, the overall performance is still very acceptable (88.42%) for the type of application we intend to develop.

## 7. Conclusions

In this work, we studied the disease detection performance of newly trained and fine-tuned CNNs on a challenging dataset that was collected in true field conditions. Data augmentation was performed to increase the number of images and make the dataset more challenging so that robust models could be trained. As we were dealing with images of trees (Plum), the images were mostly captured from the ground, resulting in differences in scales. For this purpose, we opted to study both plain CNNs as well as those architectures where multi-scale processing is performed in an integrated manner (inception networks). We observed that the inception network yielded superior performance, even in the presence of background clutter. Data augmentation resulted in achieving more robust models for disease detection. Finally, to enable efficient inference on resource-constrained devices, we quantized the Inception-v3 model from FP32 precision to FP16, gaining a 2× speedup and 2× less memory requirement.

In the future, we aim to extend this dataset further and train highly efficient object detection and semantic segmentation models for deployment on mobile devices so that fine-grained predictions can be made in an offline manner on resource-constrained devices. It will be more intuitive to indicate affected and healthy fruits individually in this manner. We also intend to make our fully annotated dataset publicly available to other researchers for use.

## Figures and Tables

**Table 1:** Network training and fine-tuning parameters.

	AlexNet		VGG-16		Inception-v1		Inception-v3	
Parameter	Train	Fine-Tune	Train	Fine-Tune	Train	Fine-Tune	Train	Fine-Tune
Input	227 × 227 × 3		224 × 224 × 3		224 × 224 × 3		299 × 299 × 3	
Batch size	128	128	8	8	32	32	16	16
Optimization Scheme	SGD-M	SGD-M	SGD-M	SGD-M	SGD-M	SGD-M	RMSProp	RMSProp
Momentum	0.9	0.9	0.9	0.9	0.9	0.9	0.9	0.9
Weight decay	0.1	0.1	0.1	0.1	0.1	0.1	0.1	0.1
Learning rate	0.01–0.0001	0.001–0.00001	0.01–0.0001	0.001–0.00001	0.01–0.0001	0.005–0.00005	0.01–0.0001	0.005–0.00005

**Table 2:** Performance of various convolutional neural networks (CNNs) newly trained on the dataset without data augmentation.

Models (Newly Trained)	Success Rate (Test)	Loss (Validation)	Epoch (Convergence)
AlexNet	53.20%	1.084	27
VGG-16	33.11%	1.420	9 (overfit)
Inception-v1	69.33%	0.956	21
Inception-v3	75.92%	0.785	25

**Table 3:** Performance of various CNNs newly trained on the dataset with data augmentation.

Models (Newly Trained)	Success Rate (Test)	Loss (Validation)	Epoch (Convergence)
AlexNet	64.06%	0.879	16
VGG-16	78.68%	0.588	13
Inception-v1	81.94%	0.535	22
Inception-v3	86.81%	0.435	28

**Table 4:** Performance of various pre-trained CNNs fine-tuned on the dataset without data augmentation.

Models (Fine-Tuned)	Success Rate (Test)	Loss (Validation)	Epoch (Convergence)
AlexNet	75.67%	0.742	8
VGG-16	82.06%	0.492	14
Inception-v1	83.12%	0.428	12
Inception-v3	86.74%	0.414	13

**Table 5:** Performance of various pre-trained CNNs fine-tuned on the dataset with data augmentation.

Models (Fine-Tuned)	Success Rate (Test)	Loss (Validation)	Epoch (Convergence)
AlexNet	81.25%	0.546	10
VGG-16	89.06%	0.292	13
Inception-v1	91.44%	0.195	12
Inception-v3	94.34%	0.146	7

**Table 6:** Confusion matrix Inception-v3 with augmented dataset.

	Brown Rot	Healthy	Nutrient Deficiency	Shot Hole	Shot Hole on Leaf	Per-Class Accuracy
**brown rot**	**96.30**	1.85	0.00	1.85	0.00	96.30%
**healthy**	2.50	**97.50**	0.00	0.00	0.00	97.50%
**nutrient deficiency**	0.00	0.00	**88.89**	0.00	11.11	88.89%
**shot hole**	4.35	0.00	0.00	**95.65**	0.00	95.65%
**shot hole on leaf**	0.00	2.08	3.00	0.00	**91.67**	91.67%

Bold values represent the True-Positives in the confusion matrix.

**Table 7:** Computational and memory requirements for various CNNs.

Model	AlexNet	VGG16	Inception-v1		Inception-v3	
Precision	FP32	FP32	FP32	FP16	FP32	FP16
Parameters	60 M	138 M	7 M		24 M	
GFLOPS	1.4	16	2		6	
Accuracy (Fine-Tuned)	81.25%	89.06%	91.44%	90.67%	94.34%	92.41%
Memory (Parameters)	233 MB	528 MB	51 MB	17 MB	91 MB	44 MB
Inference Time (ms) (SD-845)	535	1580	185	39	249	148
Inference Time (ms) (SD-801)	1530	4650	580	116	730	432
Inference Time (ms) (SD-730)	621	1900	222	48	293	173

**Table 8:** Disease detection performance on unseen samples outside the test set.

	Brown Rot	Healthy	Nutrient Deficiency	Shot Hole	Shot Hole on Leaf	Per-Class Accuracy
**brown rot**	**87.12**	7.25	0.00	5.63	0.00	87.12%
**healthy**	6.21	**90.43**	0.00	3.36	0.00	90.43%
**nutrient deficiency**	0.00	0.00	**84.04**	0.00	15.96	84.04%
**shot hole**	5.12	2.73	0.00	**92.15**	0.00	92.15%
**shot hole on leaf**	0.00	3.06	8.60	0.00	**88.34**	88.34%

Bold values represent True-Positives in the confusion matrix.
